# Up to the Herculean Task of Tackling Cancer Therapy Resistance

**DOI:** 10.3390/cancers16101826

**Published:** 2024-05-10

**Authors:** Kostas A. Papavassiliou, Athanasios G. Papavassiliou

**Affiliations:** 1 First University Department of Respiratory Medicine, ‘Sotiria’ Hospital, Medical School, National and Kapodistrian University of Athens, 11527 Athens, Greece; konpapav@med.uoa.gr; 2 Department of Biological Chemistry, Medical School, National and Kapodistrian University of Athens, 11527 Athens, Greece

Cancer therapy resistance still poses the biggest hurdle to cancer treatment. In everyday clinical practice, oncologists face resistance to numerous cancer therapies, resulting in treatment failure and poor patient survival. Due to the multifaceted nature of this major issue, overcoming cancer drug resistance demands heroic efforts from multidisciplinary research teams equipped with ingenuity, perseverance, and a large amount of funding [1].

At the molecular level, various mechanisms regarding intrinsic and acquired drug resistance in cancer have been revealed, such as reduced drug uptake or increased drug efflux, metabolic drug inactivation, genetic or epigenetic drug target alterations, upregulation of compensatory signal transduction pathways controlling cancer cell hallmarks, cancer cell plasticity, and interaction with non-cancer cells within a nurturing tumor microenvironment [2,3]. Although these mechanisms have been studied to a great extent, there are significant knowledge gaps that need to be filled.

The same challenges that stand in our way of tackling cancer drug resistance also form the opportunities we need to take advantage of them. Understanding the inherent complexity of drug resistance in cancer may be difficult, yet we can reduce its concept into a conceptual framework based on a few basic biological determinants. These include tumor growth, tumor burden, morphology and phenotype of cancer stem cells, tumor heterogeneity, physical barriers, the immune system and tumor microenvironment, undruggable oncogenic drivers, and therapeutic pressure. We can target these determinants of resistance using standard-of-care and emerging approaches as well as employing novel technological and pharmacological breakthroughs to achieve the prevention, delay, or reversal of cancer therapy resistance [4,5]. For example, in terms of the undruggable genomic drivers of cancers, the future development of gene-specific transcription factor or cofactor (coactivators, corepressors) inhibitors, compounds that restore the function of tumor suppressors, and allele-specific inhibitors will certainly boost our efforts in addressing cancer drug resistance. Along the same lines, the further development of drugs that can enhance tumor recognition by the immune system or mitigate the therapy resistance-promoting input of non-cancer cells within the tumor microenvironment will provide potent and effective therapeutic strategies.

If we are to solve the problem of cancer drug resistance, we must focus on detecting tumors at an early stage, closely monitoring therapy response and immediately adapting our therapy to resistance, achieving deeper anti-cancer responses via novel drugs and optimizing dose, schedule, and combination partners, and finally identifying cancer cell dependencies and resistance mechanisms via high-throughput screening methods that allow quick and efficient screening of samples at the model organism, cellular, pathway, or molecular level [6,7]. With the aid of machine learning and deep learning, the cancer research community will soon be ready to undertake the Herculean task of conquering cancer therapy resistance [8,9,10,11,12,13].
